# It Takes a Village: The Importance of Neuropsychological Findings in a Collaborative Approach for a Patient with Congenital Central Hypoventilation Syndrome and Specific Phobia

**DOI:** 10.1155/2021/3891481

**Published:** 2021-10-31

**Authors:** Sarah Hamill Skoch, Bo Fu, Amanda L. Stein, Samuel P. Greenstein

**Affiliations:** ^1^Department of Psychiatry and Behavioral Neuroscience, University of Cincinnati College of Medicine, Cincinnati, OH 45219, USA; ^2^Keck School of Medicine of University of Southern California (USC), Los Angeles, CA 90033, USA; ^3^Donald and Barbara Zucker School of Medicine at Hofstra/Northwell Health, Department of Psychiatry, Consultation-Liaison Psychiatry, Hempstead, NY 11549, USA

## Abstract

Congenital central hypoventilation syndrome (CCHS) is a life-threatening disorder characterized by respiratory symptoms such as hypoventilation during sleep, significantly reduced ventilatory and arousal responses, and sustained hypoxia. Patients with CCHS exhibit neurocognitive deficits due to structural abnormalities in the brainstem, cerebellum, and forebrain. Due to the potential for repeated hypoxemia and hypercarbia among patients with CCHS, neurocognitive functioning is often impaired. This is the first described report in which a patient with CCHS and specific phobia has been reported and highlights the importance of neuropsychological testing in directing treatment approaches. We report a case of a 26-year-old male, diagnosed with CCHS and specific phobia. This patient was overdue for a needed bronchoscopy to check his airway for abnormalities (recommended every 12-24 months). The patient had developed a specific phobia to procedures involving anesthesia. It was determined in the initial phase of treatment that the patient's neurocognitive status was impacting his ability to engage in psychiatric and psychosocial treatment. This patient's care consisted of neuropsychological testing, with medication consultation, and cognitive behavioral psychotherapy. Treatment involved consistent collaboration among the patient's treating clinicians as well as collaboration with the patient's family and team of nurses. At the conclusion of treatment, the patient had successfully completed his bronchoscopy and future treatment goals were identified. This case emphasizes the importance of a neuropsychological evaluation when there is a disconnect in a patient's information processing, as the results may be highly informative in directing treatment for patients with CCHS and specific phobia. The collaborative care we provided offers insights which may direct future interventions for patients with CCHS and improve their quality of life. Our case adds support to the recommendation that patients with CCHS and impaired psychosocial functioning should receive neuropsychological testing to best direct treatment.

## 1. Introduction

Congenital central hypoventilation syndrome (CCHS) is a rare genetic condition characterized by hypoventilation with progressive hypoxemia and hypercarbia during sleep but with normal ventilation while awake [1, 2]. These patients experience extreme sleep-related hypoventilation which sometimes extends to wakefulness [3]. CCHS is related to mutations of the PHOX2B gene and involves multiple autonomic nervous system disorders marked by impaired ventilatory function [3]. Respiratory arrest during sleep is a constant threat for these patients, and the majority are on long-term ventilatory support via tracheostomy. This disease has an overall high mortality rate with only 50 of 70 patients living beyond 1 year of age [4]. Additional comorbidities include cardiac conduction disorders, gastroesophageal reflux, constipation with Hirschsprung's disease, thermal dysregulation, orthostatic hypotension, ophthalmologic disorders, or neural crest tumors [5]. Patients with CCHS require ventilatory support, and a bronchoscopy is recommended every 12-24 months to examine for abnormalities in the airway [6].

In addition to the medical comorbidities, a number of neurocognitive abnormalities are present in patients with CCHS [2]. Functional magnetic resonance imaging (fMRI) studies show structural differences in the brainstem and cerebellum to the forebrain [7]. What is not clear is whether these abnormalities are caused directly by the pathology or the result of the disease-causing mutation [2]. A study conducted with children with CCHS demonstrated reduced corpus callosum fibers in motor, cognitive, speech, and ophthalmologic regulatory areas [8]. As patients advance in age, patients with CCHS evidenced decreases in the volume of gray matter in autonomic, respiratory, and cognitive regulatory areas. This change in gray matter may be a contributing factor to the functional cognitive deterioration noted in patients with CCHS [9].

**Figure 1:**
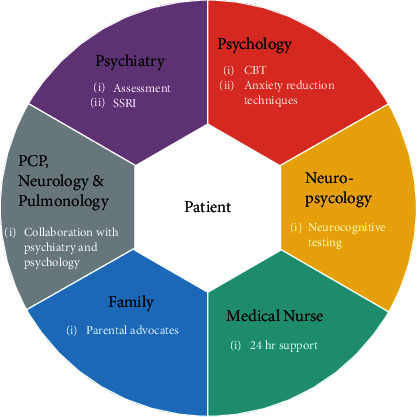
Collaborative care model of treatment for patient with CCHS and specific phobia.

Based on cross-sectional study data of 196 patients with CCHS (age range 0.4-38 years), we know that children and young adults with CCHS report a broad range of developmental, psychological, and neurocognitive problems [4]. Such problems include developmental delays (45%), learning disabilities (29.6%), attention deficit disorder (12.8%), speech delay (51%), and delays in motor development (45.4%) as discussed by Ruof et al. [4]. In the handful of studies that have been conducted, neurocognitive deficits are often present for patients with CCHS as well as impaired psychosocial functioning [10]. Although the symptoms and resulting impairments caused by CCHS may lead to significant anxiety, few published reports exist of this association. Even fewer published studies discuss treatment options for psychiatric management of cognitive and psychiatric symptoms for patients with CCHS. Due to the rarity of CCHS, many physicians are unaware of this diagnosis and may only treat one of these highly specialized patients over the course of their professional work [11]. The role of neuropsychological evaluation was critical in our understanding this patient's current functioning and to direct our treatment approach. Therefore, we provide the details of this case as well as the highly collaborative nature (see Figure 1) of the care provided in an effort to both increase awareness about this syndrome and share key components of treatment that may be applied to other individuals with CCHS and psychiatric comorbidities.

## 2. Case Presentation

Our patient, heretofore referred to as “Mr. A,” was a 26-year-old male diagnosed with CCHS, generalized convulsive epilepsy, and a history of a nonverbal processing disorder. Mr. A was diagnosed with CCHS at birth, and his development was greatly impacted by this diagnosis. Mr. A was ventilator dependent 16-18 hours per day. In addition, Mr. A was diagnosed with epilepsy as a teenager and was on antiepileptic medications with mood-stabilizing benefits including divalproex 250 mg every morning and 500 mg every night and lamotrigine 400 mg every night. He was also on clonidine 0.3 mg patch every three days for hypertension and hyperactivity. Psychiatric interview performed by the authors determined diagnoses of specific phobia (blood-injection injury subtype), along with history of attention deficit hyperactivity disorder and history of nonverbal learning disorder. We administered a Beck Depression Inventory (BDI) at intake, and the patient scored in the “minimal” range on this measure. As depression is frequently comorbid with chronic medical illness, it is possible that the patient experienced underlying depressive symptoms that were not captured at intake, as the treating psychiatrist later diagnosed him with unspecified depression.

At the time he presented for intake, he and his family reported poorly controlled anxiety symptoms that were interfering with his quality of life and medical management of his care. Specifically, it had been 26 months since his last bronchoscopy and Mr. A was refusing this procedure due to fears he had developed about anesthesia. Mr. A had completed over 20 bronchoscopies throughout his life, and yet in the preceding few months, he developed a phobia to the anesthesia used in these procedures. We hypothesized that this phobia was due to his growing and yet perhaps limited understanding of the pathophysiology of his own illness. Mr. A expressed worries that he would stop breathing and never wake up and became anxious about completing future procedures. His pulmonology team recommended he receive bronchoscopies every 12-24 months to check for polyps and air leakage from his ventilator, as well as airway abnormalities. Mr. A and his family were referred to treatment with the goal of reducing his anxiety related to these procedures.

Regarding mental status exam and based on psychiatric interview performed by the clinical psychologist, psychiatrist, and neuropsychologist, Mr. A denied symptoms of depression, mania, generalized anxiety about everyday life, trauma, or psychosis. He had no prior history of mental health services either with therapy or medication management by a psychiatrist. Mr. A had no history of inpatient psychiatric hospitalizations, nor did he have history of self-injurious behavior or suicide attempts/suicidal ideation. He denied all substance use, including alcohol or tobacco. Family history was positive for anxiety and depression.

Neuropsychological history was significant for academic and cognitive deficits. Our treatment team had questions regarding Mr. A's ability to understand and synthesize medical information from the various treatment providers. Further developmental and cognitive history was obtained by the psychologist and psychiatrist. Mr. A had a history of delayed developmental milestones including walking (age 17 months) and talking (age 4 years), as well as difficulty with reading comprehension. He underwent neurocognitive evaluation at distinct time points during childhood. His test performance at age 16 was markedly worse in several domains compared with his performance at age 9, including visual-spatial function, processing speed, attention, and executive function. These findings were interpreted to reflect a nonverbal learning disorder. Mr. A received special education services in reading and writing while in high school and attended online courses during the summer. He received As, Bs, and Cs while in high school and went on to complete a four-year bachelor's degree in liberal arts. Notably, it was reported that his mother provided substantial assistance during college. Mr. A had difficulty with oral expression and receptive language/comprehension. It was hard for him to see “shades of gray” or interpret other people's reactions. His nursing staff reported that Mr. A's inattention was causing him difficulty in applying for jobs. There was concern about additional cognitive decrement since his most recent evaluation at age 16, and referral was placed for repeat neuropsychological testing to better direct treatment.

Treatment for Mr. A consisted of obtaining neuropsychological testing and reviewing the results of this testing with the patient's treatment team and family. This was performed by one of the coauthors, a trained neuropsychologist. Results from this testing was then used in directing his treatment which included (1) psychiatry medication consultation and follow-up and (2) psychotherapy for his specific phobia and anxiety symptoms. Feedback was provided to the family at periodic intervals, as well as a joint meeting between the family, clinical psychologist, neuropsychologist, and treating psychiatrist to review the findings of neuropsychological testing and how those findings would be useful in directing treatment. The emphasis of treatment was patient-centered care with consistent collaboration between medical teams (neurology/pulmonology), psychiatry team (psychiatry, psychology, and neuropsychology), and the patient's family and nursing staff (see Figure 1).

### 2.1. Neuropsychological Testing

The neuropsychological testing documented moderate to severe cognitive deficits in cognitive domains including attention, executive ability, language and social communication, and learning (see Table 1). Attentional limitations included reduced immediate memory span capacity and inconsistency in his ability to maintain focus of attention over time. Severe executive deficits included disorganization, trouble with gestalt organization and integration, and poor response regulation. Mr. A focused on details and processed information in a fragmented manner, which contributed to trouble appreciating relationships and contextual cues. He had difficulty grasping abstract concepts or to draw inferences and tended to be cognitively inflexible; accordingly, his approach to tasks was concrete, and he exhibited conceptual confusion with nonliteral, abstract concepts. This affected problem-solving as well as comprehension and learning.

He exhibited deficits in both spoken language and reading, with relatively greater impairment for oral and aural language as compared with written language. He struggled with verbal and nonverbal aspects of interpersonal communication; these deficits interfere with social efficacy. Learning and memory problems were apparent, largely secondary to attention and executive deficits that disrupted effective memory acquisition, encoding, and retrieval processes. This was most evident on more demanding learning tasks involving complex material, where he struggled to organize and integrate information effectively.

Compared with prior evaluations, the findings were largely comparable, with no indication of significant improvement in any domain. The nature and extent of cognitive dysfunction confirmed he would continue to require assistance with various aspects of everyday life, although certain interventions were recommended to improve function and quality of life. Organizational strategies were suggested, given the level of cognitive disorganization. Repetition and visual aids were identified as strategies that could help improve comprehension and skill acquisition. It was suggested that Mr. A be accompanied to all medical appointments to assist with medical decision-making. Ultimately, guardianship or another legal designation might be pursued, depending on his clinical and functional course.

Notably, Mr. A expressed a strong desire to engage in productive work and to perform well. In addition, despite interpersonal communication challenges, he presented as friendly and personable. Cognitive dysfunction is such that he would require individualized instruction and ongoing support at any job to develop and maintain occupational skills. In addition, he would be best suited for a job involving simple, routine tasks that he could master.

### 2.2. Medication Consultation and Consultation

During the course of treatment, the treating psychiatrist met with and evaluated the patient. Mr. A was trialed on sertraline as a treatment for anxiety, but the patient had a seizure a few days after starting it. The treating psychiatrist felt that sertraline was unlikely to be the culprit of the patient's seizure, and while the primary neurologist concurred that sertraline likely was not the culprit of the seizure, out of an abundance caution, this medication was discontinued. Medication follow-up and consultation occurred throughout the case. The patient's psychiatrist provided highly collaborative care by periodic interdisciplinary telephone calls with the patient's primary neurologist, who had worked with Mr. A for more than 10 years. The treating psychiatrist further communicated the patient's neuropsychological findings, with patient and family consent, to promote more effective medical care. This patient often struggled to understand recommendations from his neurologist, and presentation of the neuropsychological findings enhanced the medical team's understanding of the best way to present information to this patient and his support team. While a medication for anxiety was not pursued, Mr. A and his family discussed the benefits they received by the psychiatrist's follow-up, supportive therapy provided, and collaboration with neurology. The treating psychiatrist facilitated a discussion of the findings from the treatment team and neuropsychological findings. In this meeting, the treatment team reviewed findings from the neuropsychological report, medication management, and patient's response to psychotherapy and medication.

### 2.3. Psychotherapy for Anxiety and Specific Phobia

Using data from aforementioned neuropsychological testing, strategies were employed in treatment to enhance communication. These strategies included visual aids, repetition, and reinforcement of information. This was done to allow for enhanced communication due to the interactive complexity of Mr. A's expressive and comprehensive language deficits, as well as to include his caregivers in treatment as a way to increase consistency and application of therapy practices in between sessions. The clinical psychologist engaged Mr. A in cognitive behavioral anxiety reduction strategies during individual psychotherapy. Treatment consisted of a modular approach over the course of six months, incorporating relaxation training, coping strategies for anxiety reduction, and exposure therapy to reduce patient's fear regarding bronchoscopy. Exposure therapy included *in vivo* exposures in session to this feared medical procedures, as well as between session exposure practices such as viewing photographs of bronchoscopy procedures, visiting the office/hospital where the bronchoscopy would take place, and scheduling several planned pre-op visits to habituate to the OR setting where the procedure would take place. Psychotherapy visits included either Mr. A's mother or a member of his nursing staff.

## 3. Discussion

Treatment for this patient involved consistent collaboration across multiple disciplines including the patient's medical teams, nurses, and family. Due to the family's concern for increased risk of seizure, nonpharmacologic interventions (i.e., cognitive behavioral therapy) became the primary avenue for treatment. Concerns about the patient's communication, comprehension, and decision-making abilities became apparent. The decision was made to obtain repeat neuropsychological testing which documented moderate to severe cognitive impairments in attention, executive ability, language, and learning. The nature of the findings and the available history suggested the deficits reflected acquired, neurocognitive impairment related to seizure disorder and cerebral hypoxia associated with CCHS. His recent neuropsychological testing provided further clarity into his difficulty with processing medical information as well as focus in our treatment approach. By utilizing neuropsychological recommendations to direct treatment, at treatment end, the patient successfully completed his bronchoscopy, and anxiety symptoms were reduced. Continued goals for ongoing therapy included further developing his independent living skills and interpersonal functioning, as neuropsychological testing documented that these deficits were evident. The present case report contributes to this literature on CCHS, as few published reports exist on psychiatric and psychological treatment options for these complex patients.

CCHS is a rare condition with prevalence rates estimated to be 1/500,000 individuals [5]. Hallmarks of this disease include global autonomic dysfunction such as Hirschsprung's disease and neural crest tumors and absent ventilatory responses to hypercapnia and hypoxia [5]. These patients often require 24/7 care, ventilator support, and a team of medical and psychological specialists. Many patients with CCHS demonstrate both cognitive disruptions and anxiety/mood lability [1]. CCHS typically presents in the neonatal period and is characterized by hypoventilation, autonomic dysregulation, and elevated risk for neurocognitive dysfunction [12].

Emerging research supports the need for comprehensive neuropsychological evaluation in patients with CCHS [10]. On average, patients with CCHS have demonstrated intellectual functioning scores that are estimated to be in the borderline impaired range. However, previous work also shows there can be a wide range of IQ scores [10]. Several studies have demonstrated that individuals with CCHS demonstrate average coping skills and behavioral difficulties—suggesting that many individuals with CCHS are resilient and have the ability to cope with stressors [13]. Due to the significant variability in cognitive functioning and coping abilities, a growing body of research as well as the America Thoracic Society suggests that all patients with CCHS receive a neuropsychological evaluation to document strengths and weaknesses as well as provide insight into recommendations for treatment [10, 14].

## 4. Conclusions

A diagnosis of CCHS results in considerable burden for families due to the physical care and daily stress of managing an individual with this illness, as well as concerns about the individual's well-being [3]. Patients with CCHS have a variety of medical and psychological challenges and require a great deal of specialized medical care. Importantly, emerging evidence supports the fact that CCHS is associated with poor neurocognitive outcomes [10]. Research has demonstrated that the recurrent hypoxia that occurs primarily in the first few years of life plays a significant role in brain development and brain imaging studies have shown that certain brain structures with known involvement in neurocognition are smaller in CCHS patients when compared to controls [5]. Few published reports on patients with CCHS and anxiety exist, and even fewer have documented ways in which to assess neurocognitive functioning as a way to direct and focus treatment for medical and psychiatric providers.

This case contributes to the limited literature published of collaborative care in CCHS patients with anxiety about medical procedures. The treatment approach in this case emphasizes the importance of neuropsychological evaluation and management of neuropsychological deficits that may impact care in CCHS patients. CCHS patients may acquire neuropsychological deficits that further affect their quality of life [7]. Neuropsychological testing for our patient provided information and results that allowed us to better target psychotherapy sessions, tailor the cognitive-behavioral therapy, psychiatric care, and improve the patient's understanding of his medical condition and the rationale for completing his bronchoscopy.

This case also highlights the significant benefit of collaborative care within psychiatry and psychology, particularly for patients with complex medical diagnoses and comorbid anxiety. While excellent support exists for the effectiveness of collaborative care models for certain conditions (cancer, heart disease, and pain), few published reports exist on how to apply collaborative care to CCHS patients [15]. While we know collaborative care promotes positive health outcomes for patients, such models of collaborative care can be challenging to execute.

Additional research is needed to examine how neuropsychological functioning can best inform medical, psychiatric, and psychological interventions for patients with CCHS with the goal of improving the patient's quality of life. Longitudinal data is needed to document long-term follow-up of neuropsychological functioning in this population. Future studies are also needed to examine protective factors in this population such as social skills and coping mechanisms that might promote resilience in patients with CCHS as well as improve medical outcomes.

## Figures and Tables

**Table 1:** Results of neuropsychological testing percentiles.

Test	Percentiles
WAIS-III: picture completion	0.4^th^
WAIS-III: block design	1^st^
WAIS-III: comprehension	16^th^
WAIS-III: object assembly	0.1^st^
Auditory verbal sequencing span	7^th^
CORSI (visual-nonverbal sustained attention)	3^rd^
Porteus maze test	<0.1^st^
California verbal learning test (learning trials)	7^th^
Word comprehension subtest	1^st^
Benton word fluency	0.1^st^

## Data Availability

Data is not freely available due to patient privacy issues, and the data presented in this report was anonymized to protect privacy. Written informed consent was obtained from the patient for publication of this case report and the accompanying data. A copy of the written consent is available for review by the Editor-in-Chief of this journal.
